# Responsibility of education in improving medical college students’ ability to prevent and respond to public health emergencies in China – A systematic review

**DOI:** 10.3389/fpubh.2023.1191723

**Published:** 2023-11-21

**Authors:** Xin Fang, Lei Zhao, Ran Pang, Huarong Li, Pian Ye

**Affiliations:** ^1^Department of Geriatrics, Union Hospital, Tongji Medical College, Huazhong University of Science and Technology, Wuhan, China; ^2^Department of infectious Disease, Union Hospital, Tongji Medical College, Huazhong University of Science and Technology, Wuhan, China; ^3^Department of Integrated Traditional Chinese and Western Medicine, Union Hospital, Tongji Medical College, Huazhong University of Science and Technology, Wuhan, China

**Keywords:** responsibility of education, medical education, medical college students, public health emergencies, prevention, response

## Abstract

**Background:**

The outbreak of coronavirus disease 2019 (COVID-19) has highlighted the critical importance of sufficient preparedness for public health emergencies. This places higher requirements on the ability of medical staff to deal with such emergencies. Nonetheless, education courses on public health emergencies in China are usually aimed at public health students, and not at all medical college students. Importantly, these medical students will become medical workers who are generally the first-contact personnel and play an irreplaceable role in responding to most public health emergencies. Therefore, it is urgent to strengthen educational courses to enable these students to adequately prevent and respond to public health emergencies.

**Objectives:**

The purpose of this systematic review was to reveal the current unsatisfactory status of Chinese medical college students’ knowledge and skills in dealing with public health emergencies and their training needs.

**Methods:**

We searched EMBASE, PubMed, Google Scholar, Web of Science, CNKI, Wan Fang, and VIP Information Network for all associated original studies written in English and Chinese from the inception of these databases until March 12, 2022.

**Results:**

This systematic review screened out 15 eligible studies that met the inclusion criteria. These studies demonstrated that Chinese medical college students generally have a low ability to deal with public health emergencies. Most students believe it is essential to master coping with public health emergencies and desire to acquire this knowledge. But the participation rate is low, and only a few students actively seek relevant knowledge.

**Conclusion:**

The findings of this review illustrate the importance of improving medical college students’ education to prevent and deal with public health emergencies. It is necessary to improve medical college students’ education in responding to public health emergencies.

**Systematic Review Registration:** PROSPERO, Identifier [CRD42023467374].

## Introduction

The COVID-19 pandemic has brought worldwide attention to preventing and responding to public health emergencies. Dealing with the pandemic has exposed the lack of competent medical talent to handle public health emergencies and revealed the drawbacks of the separation between clinical medicine and public health or preventive medicine in current medical education (1).

Public health emergencies refer to sudden outbreaks of major infectious diseases, mass illnesses of unknown origin, major food or occupational poisoning, and other events that seriously affect public health and cause or may cause severe damage to the health of the population (2). These events easily trigger public panic, anxiety, and other emotions, and their abruptness and collective nature have substantial negative impacts on the population, politics, economy, trade, and human health, among others (3, 4). Effective responses are crucial to minimizing the adverse impact of public health emergencies.

In recent years, the world has been facing an increasing number of public health emergencies. With the development of science and technology as well as societal progress, communication among different countries or regions is becoming more frequent and closer. Concurrently, the scale and complexity of public health emergencies are becoming more severe. Public health emergencies are not limited to a single country, but can spread rapidly to neighboring countries and even around the world in various ways. According to information released on the website of the National Health Commission of the People’s Republic of China, among all kinds of public health emergencies, those involving infectious diseases are the most common and serious, followed by food poisoning, occupational poisoning, and environmentally related events (5). These events have challenged the capacity of many countries, especially developing countries, to prepare for and respond to public health emergencies.

Public health emergencies are difficult to predict and cannot always be avoided, as they can occur in different forms in the future. Therefore, optimizing prevention and control is of the utmost importance. Medical workers are the first contact personnel in most public health emergencies. They play crucial roles in responding to public health emergencies and are the main force in prevention and control (6).

In ongoing public health emergencies, however, the coping ability of medical workers is a concern and can reveal problems between clinical medicine and public health and preventive medicine in China as well as in other countries (6). For example, during the severe acute respiratory syndrome (SARS) epidemic in 2003 as well as the severe acute respiratory syndrome coronavirus 2 (SARS-CoV-2) outbreak in 2019, medical workers were among the group of people experiencing the highest levels of stress and anxiety (7, 8). The infection rate of these medical staff was the highest among all groups, and the rate of infection was very high during the initial stages of these outbreaks (6, 7, 9, 10). In addition, severe nosocomial infections can occur in hospitals owing to nonstandard disinfection and isolation measures during a public health emergency (6, 7, 9). This situation can lead to environmental exposure of medical personnel. Many of the health professionals affected have insufficient ability to deal with a public health emergency, highlighting the importance of these medical personnel mastering the knowledge and skills to prevent and respond to public health emergencies (7).

Medical education is the cornerstone of the medical and health industry. To some extent, the problems exposed by public health emergencies expose the defects and deficiencies in medical education, which is focused on clinical medicine and neglects public health prevention (6, 11, 12). Because in China and some other countries, clinical medicine and public health/preventive medicine are taught separately. Medical students have a nearly exclusive focus on individual patients during their training. Therefore, students became proficient in the micro-dynamics of disease and its treatment, but risk losing their ability to see the patient’s context and reasons behind their illness, such as unhealthy behaviors. Medical students rarely receive education on how public health might be relevant to their clinical careers (1). Many medical schools remain solely responsible for medical students’ curricula and education and are mostly disconnected from the field of public health (13). Even most educational programs designed to prepare healthcare professionals are not organized to accommodate the rapid and often unpredictable changes in public health emergencies (13). In addition, medical colleges must equip their students with the skills to interact within multidisciplinary teams. However, to our knowledge, none of the existing curricula represent a systematic effort to prepare a large cadre of health professionals to respond to public health emergencies using a multidisciplinary, interdisciplinary, and collaborative approach (14). Finally, the number of instructors in public health education is insufficient, the educational structure is defective, and their teaching capacity is limited (15).

We sought to understand the knowledge and skill level of medical college students in dealing with public health emergencies and their training needs and to identify weak points in the relevant knowledge of these students in the previous education process in China. Our findings can help in achieving targeted and practical education for future medical college students in responding to public health emergencies and integrating clinical medicine and public health and preventive medicine education.

Medical graduates increasingly need public health skills to equip them to face the challenges of health care practice in the 21st century (16). Integrating clinical medicine with public health and preventive medicine in the course of medical education reform is particularly important for a timely and effective response to public health emergencies. However, to date, there has been no relevant literature analyzing the current situation and systematically discussing this issue. In this paper, we aim to fill this gap. We systematically analyzed medical students’ cognitive status and training needs in responding to public health emergencies, identified existing problems and shortcomings, and then proposed possible solutions.

## Methodology

### Literature review

We performed this study in accordance with guidelines outlined in the Preferred Reporting Items for Systematic Reviews and Meta-Analyses (PRISMA) (PROSPERO ID: 467374) (17). We conducted a comprehensive search for relevant studies (written in English and Chinese) from major online databases, such as EMBASE, PubMed, Google Scholar, Web of Science, CNKI, Wan Fang, and VIP Information Network, from the construction of these databases to 12 March 2022. Two independent reviewers scanned the literature and included the eligible studies by common consensus after multiple rounds of screening.

### Data sources and search methods

**Figure 1:**
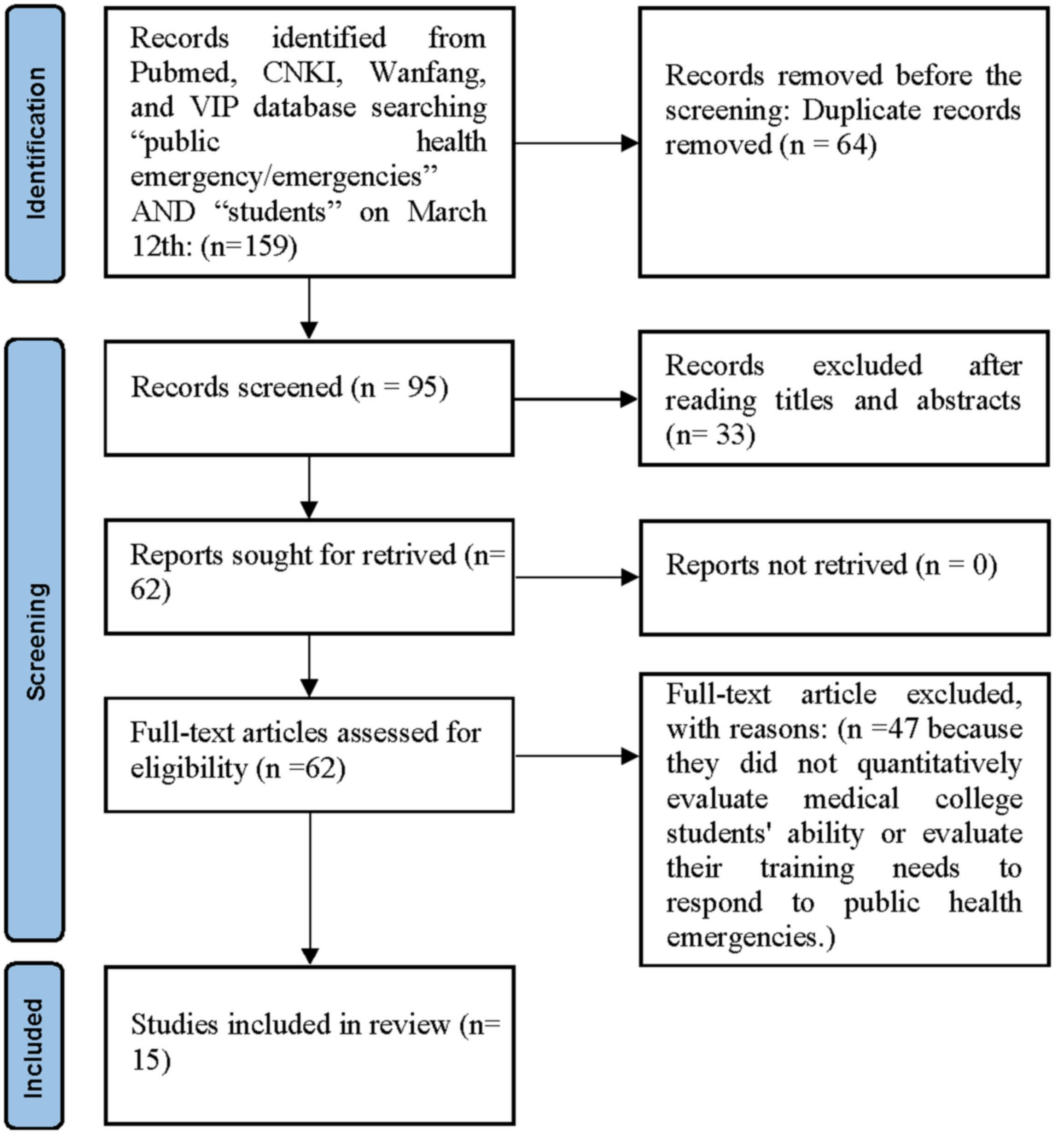
Articles assessment diagram.

The search process included (i) reading the reference section of all relevant research carefully; and (ii) manually searching abstracts of key journals and papers published at major annual conferences. The search terms used were a mix of (“students” [Title] AND “public health emergency” [Title]) OR (“students” [Title] AND “public health emergencies” [Title]). We also checked the reference lists of the screened studies to identify other similar studies. The search strategy is shown in Figure 1. We included studies of medical college students’ knowledge and training needs for responding to public health emergencies. The PICOS criteria are used to select the eligible studies. Studies were included if they satisfied the following inclusion criteria: (i) the study was limited to original empirical study and humans; (ii) the study was conducted in Mainland China; (iii) the study was peer-reviewed article with full-text available; (iv) all participants are medical college students and need to include clinical medicine majors; (v) the study was written in English or Chinese; (vi) the study quantitatively reported medical college students’ knowledge or training needs for responding to public health emergencies. The exclusion criteria were as follows: (i) the studies focused on non-medical college students or clinical medicine students were not involved; (ii) the study conducted in other regions/countries rather than Mainland China; (iii) meeting abstracts, reports, editorials, or reviews; (iv) unavailable fulltext articles; (v) the study did not quantitatively evaluate medical college students’ ability or evaluate their training needs to respond to public health emergencies; (vi) duplicated reports.

### Data extraction

For all articles included, we extracted the following information from the original articles using a standardized form, including the relevant data about bibliographic details: (first author, publication year), participant characteristics (sample size, University/college placement), and outcomes.

### Quality assessment

As the reviewed articles differed in research design, a quality assessment tool developed by Rowe et al. that has been proven to be a useful tool for assessing qualitative, quantitative, and mixed methods was utilized (18, 19). The tool assesses five important methodological aspects of a study, namely the background or literature review, sample, study design or methodology, outcome measures, and conclusions (20). The total score ranges from 0 to 5, with the higher scores representing better methodological quality. Articles scoring 4 or 5 are considered to be high in quality, articles scoring 3 are considered to be of moderate quality, and studies scoring between 0 and 2 are considered to be low in quality. In this review, all included studies were independently evaluated by two reviewers (XF and PY). Divergences were resolved through discussion and consensus. Discrepancies between the two reviewers were resolved through discussion with a third author until consensus was finally reached. All 15 articles received a score between 4 and 5, indicating their high methodological quality.

## Results

### Study characteristics

The results of the systematic review are presented in Figure 1. We identified a total of 15 studies related to the cognitive status and training needs of medical college students in response to public health emergencies in China after a thorough review of all papers. The characteristics of the studies are listed in Table 1. Among the studies considered in this paper, all were conducted in China.

**Table 1:** Characteristics of included studies.

References	Year	Data collection location	Sampling method	Evaluation method	Participants	Sample size	Mean age	Gender (M/F)
Hu et al. (21)	2004	Health Science Centre of Lanzhou	Cluster sampling	Self-administered spot field questionnaire survey	Newly graduated medical students	463	NA	NA
Yu et al. (22)	2011	Zhengzhou University	Stratified cluster sampling	Self-administered spot field questionnaire survey	Medical college students in their first-fifth year	460	NA	NA
Zheng et al. (23)	2012	Putian University	Cluster sampling	Self-administered on-site anonymous questionnaire survey	Undergraduate medical students; junior medical college students	332	NA	NA
Wang et al. (24)	2012	Five medical colleges in Guizhou Province	Random sampling	Self-administered spot field questionnaire survey	Medical students of different majors and genders in grades 1 to 4	2,290	NA	925/1365
Liu et al. (25)	2013	Hainan Medical University	Multi-stage random sampling	Self-administered self-filling questionnaire survey	Undergraduate medical students; junior medical college students	1,276	NA	524/752
Zhao et al. (26)	2013	Lanzhou University and Gansu University of Chinese Medicine	Cluster random sampling	Self-administered on-site anonymous questionnaire survey	Undergraduate medical students	451	20 ± 0.61	285/166
Zheng et al. (27)	2015	Changsha Medical College	Cluster random sampling	Self-administered KAP questionnaire survey	First-third year medical college students	506	NA	NA
Mu et al. (28)	2015	Five colleges and universities in Jilin City	Stratified cluster sampling	Self-administered on-site anonymous self-filling questionnaire survey	First-fourth-year medical college students	71	NA	NA
Wu et al. (6)	2015	China Medical University	Cluster sampling	Self-administered on-site anonymous questionnaire survey	2009 medical students majoring in clinical and preventive medicine	176	22.25	102/136
Liu et al. (29)	2017	Five universities in Hainan Province	Multi-stage random sampling	Self-administered self-filling questionnaire survey	Undergraduate medical students; junior medical college students	1,100	NA	NA
Liu et al. (30)	2018	Three universities in Wuhu City, Anhui Province	Stratified random cluster sampling	Self-administered on-site KAP questionnaire survey	Medical college students in their first-third year	381	NA	NA
Hu et al. (31)	2020	Health Science Center of Shengzhen University	Cluster sampling	Questionnaire survey	2018 clinical medical students	58	NA	NA
Sun et al. (12)	2021	Binzhou Medical University	Convenience sampling	Network anonymous questionnaire survey	Medical college students in their first-fifth year	366	NA	141/225
Cao et al. (32)	2021	Six universities in Shandong Province	Stratified random sampling	Self-administered network anonymous questionnaire survey	Medical college students in their first-fifth year	2,153	NA	NA
Tang et al. (33)	2022	Southwest Medical University	Convenience sampling	Network questionnaire survey	Medical college students in their first-fifth year	5,465	NA	1676/3789

### Quality assessment

Table 2 shows the results of a methodological quality assessment of all included studies. Due to the degree of heterogeneity observed in the study design, and outcome indices, meta-analysis was considered impractical. 15 studies mainly used a quantitative research design (6, 12, 21–33). All 15 articles received a score between 4 and 5, indicating their high methodological quality. All studies adopted a questionnaire survey as the significant approach to collect data. 3 studies provided unclear information of participants and participants in 2 studies were recruited through convenience sampling.

**Table 2:** Methodological quality assessment of the included studies.

References	Year	Background/Literature review	Sample	Study design or methodology	Outcome measures	Conclusions	Total score	Methodological quality
Hu et al. (21)	2004	1	0	1	1	1	4	High
Yu et al. (22)	2011	1	1	1	1	1	5	High
Zheng et al. (23)	2012	1	0	1	1	1	4	High
Wang et al. (24)	2012	1	1	1	1	1	5	High
Liu et al. (25)	2013	1	1	1	1	1	5	High
Zhao et al. (26)	2013	1	1	1	1	1	5	High
Zheng et al. (27)	2015	1	1	1	1	1	5	High
Mu et al. (28)	2015	1	1	1	1	1	5	High
Wu et al. (6)	2015	1	1	1	1	1	5	High
Liu et al. (29)	2017	1	1	1	1	1	5	High
Liu et al. (30)	2018	1	1	1	1	1	5	High
Hu et al. (31)	2020	1	0	1	1	1	4	High
Sun et al. (12)	2021	1	1	1	1	1	5	High
Cao et al. (32)	2021	1	1	1	1	1	5	High
Tang et al. (33)	2022	1	1	1	1	1	5	High

### Cognitive status of medical college students in response to public health emergencies in China

As shown in Table 3, in the 15 studies, questionnaire surveys on medical college students’ knowledge and training needs in responding to public health emergencies were conducted. The average score representing medical college students’ knowledge of coping with public health emergencies only ranged from 52.13 ± 8.17 to 79.43 ± 10.40 (the total score was uniformly converted to 100 points). The percentage representing the passing rate of medical college students in the assessment of coping with public health emergencies, or the awareness/mastery/accuracy rate of knowledge related to public health emergencies, ranged from 32.55 to 72.38% (6, 12, 25, 33) (Table 3). These studies demonstrated that respondents generally have a low ability to deal with public health emergencies. Zhao further showed that these students self-evaluated their emergency response ability highly (data not shown) (26).

**Table 3:** Results of included studies.

Author and year	Number (percentage) of students	Number (percentage) of students	Average score (*n* = Medical college students)	Passing/Awareness/Mastery/Accuracy rate (*n* = medical college students)
Hu et al. (21)	–	–	–	53.33% awareness rate, *n* = 463
Yu et al. (22)	1061 (88.80%) hoped to acquire relevant knowledge of public health emergencies further	–	78.56 ± 9.90, *n* = 460	–
Zheng et al. (23)	–	–	52.13 ± 8.17, *n* = 332	–
Wang et al. (24)	1884 (82.2%) willing to receive training in public health emergencies	–	–	–
Liu et al. (25)	80.0% of students willing to learn about public health emergencies and think it is necessary to set up relevant courses and conduct emergency drills.	–	65.86 ± 13.96, *n* = 1276	71.54% awareness rate, *n* = 1276
Zhao et al. (26)	388 (86%) think it is necessary to set up relevant elective courses in medical colleges	207 (46%) believe that disasters are far removed	–	–
Zheng et al. (27)	–	–	77.23 ± 13.11, *n* = 506	–
Mu et al. (28)	248 (85.50%) hope to acquire further knowledge related to public health emergencies and the coping methods	–	–	50% awareness rate, *n* = 71
Wu et al. (6)	169 (96.6%) felt it was necessary for medical students majoring in non–preventive medicine to master the relevant knowledge of public health emergencies	–	–	72.38% awareness rate, *n* = 176
Liu et al. (29)	1684 (82.59%) thought it was important to master the knowledge of public health emergencies; 1532 (75.14%) thought colleges needed to carry out relevant teaching activities	795 (38.99%) would take the initiative to seek knowledge about public health emergencies	69.18 ± 16.56, *n* = 1100	–
Liu et al. (30)	830 (77.7%) thought it was necessary to popularize the knowledge of public health emergencies and relevant emergency training	–	57.62 ± 9.30, *n* = 381	–
Hu et al. (31)		13 (22%) actively sought information about public health emergencies	–	67% awareness rate of public health emergencies; 45% mastery rate of knowledge on handling public health emergencies, *n* = 58
Sun et al. (12)	348 (95.00%) believed that it was necessary to conduct training and exercises related to public health emergency response	193 (52.73%) participated in training related to coping with public health emergencies	54.28 ± 15.40, *n* = 366	46.99% passing rate, *n* = 366
Cao et al. (32)				66.28% passing rate, *n* = 2153
Tang et al. (33)	5244 (96%) believed that medical college students needed to carry out training in knowledge and skills of emergency handling of public health emergencies			32.55% average accuracy rate of public health emergency knowledge, *n* = 5465

The above studies showing the number (ratio) of students who thought it was essential to master the knowledge of public health emergencies. They hoped to acquire the related expertise further and believed colleges should carry out relevant emergency training and public health emergency response exercises. Individual studies also show the number (rate) of students who would take the initiative to seek knowledge about public health emergencies and participate in the training related to coping with public health emergencies. One study showed that many students believed that disasters were far from themselves. In the above studies, some questionnaire surveys were conducted on their ability to respond to public health emergencies among college students in several universities. The average score represents medical college students' knowledge of coping with public health emergencies. The percentage represents the passing rate of medical college students dealing with public health emergencies or the awareness/mastery rate of expertise related to public health emergencies.

### Training needs of medical college students in response to public health emergencies in China

The results of these studies showed that the majority of college students thought it was essential to master knowledge regarding public health emergencies (82.59%), hoped to acquire further related expertise (82.2–88.80%), and believed that it was necessary for colleges to carry out relevant emergency training and exercises in public health emergency response (75.14–96.6%) (6, 12, 24–26, 28–30, 33). However, not many college students would take the initiative to seek knowledge about public health emergencies (38.99%), participate in training related to coping with public health emergencies (52.73%), or actively seek information on public health emergencies (22%) (12, 29, 31). Zhao showed that many medical college students (46%) believed disasters were far removed from their daily life (26).

In Table 4, four of the 15 studies demonstrated that medical college students’ expectations in public health emergencies were diverse in their choice of education methods (12, 21, 26, 29). The preferred training methods include watching media or video materials (1,232 students, 37.12%), using the Internet (1,164 students, 35.07%), and lectures (752 students, 22.66%).

**Table 4:** The following four studies show the preferred training methods of medical college students in knowledge or skills related to public health emergencies.

Author and year	University	Training methods	Number (Composition ratio) or frequency
Hu et al. (21)	Health Science Centre of Lanzhou University	1. Media (TV) 2. Radio 3. Special lectures on health education 4. Newspapers and books 5. Internet 6. Communication with insiders via telephone 7. Hotline	403 (87.04%) 361 (77.97%) 317 (68.47%) 314 (67.82%) 261 (56.37%) 122 (26.35%) 113 (24.41%)
Zhao et al. (26)	Lanzhou University and Gansu University of Chinese Medicine	1. Educational media 2. Visiting popular science education bases 3. Lectures 4. Contests with prizes	192 (42.57%) 164 (36.36%) 159 (35.25%) 141 (31.26%)
Liu et al. (29)	Five universities in Hainan Province	1. Network 2. Television 3. School teaching	768 (37.67%) 419 (20.55%) 376 (18.44%)
Sun et al. (12)	Binzhou Medical University	1. On-site demonstration 2. Attending lectures 3. Watching video materials 4. Learning relevant elective courses on campus 5. Using a first-aid knowledge exchange platform 6. Reading professional books 7. Reading brochures	304 (83.06%) 276 (75.41%) 218 (59.56%) 184 (50.27%) 135 (36.89%) 125 (34.15%) 103 (28.14%)

From the above results, it can be seen that students in medical colleges have high self-evaluation of their ability to respond in a public health emergency. However, they do not have a comprehensive grasp of knowledge regarding coping skills, their cognitive level is not high, their practical knowledge is weak, and their crisis awareness needs further improvement. On the one hand, most students believe it is essential to master coping with public health emergencies and desire to acquire this knowledge. They believe that colleges must offer relevant courses and conduct public health emergency drills, indicating that these students wish to strengthen their knowledge and skills training. On the other hand, the participation rate is low, and only a few students actively seek relevant knowledge (6, 12, 21–23, 25–33).

## Discussion

### A brief introduction to the model of public health education of clinical medicine in China

At present, the training mode of undergraduate education in the clinical medicine specialty in Chinese higher medical colleges adopts the former Soviet Union education mode, with clinical and public health education separated from each other (34). The emphasis on public health education is insufficient, and the education funds and teaching investment are relatively low (35). Although public health education runs through the whole process of clinical medical education, there is less cross-teaching between public health education and clinical medicine, and public health education is not closely related to clinical practice (36). The curriculum is basically “compulsory + elective,” all of which are held by teachers from the College of Public Health (34). The public health teaching materials mainly focus on courses such as epidemiology, health statistics, labor hygiene, and environmental hygiene, and the content is disconnected from clinical knowledge, resulting in low enthusiasm, insufficient attention, and a sense of mission for students to learn public health (35).Due to the constraints of traditional teaching methods such as syllabus requirements, class hour limitations, and exam methods, most universities adopt a cramming teaching method where teachers give lectures and theoretical teaching is the main focus. Students have low levels of participation and low learning enthusiasm.

In class, more emphasis is placed on the teaching of subject knowledge and the training of basic skills. Social field practice and case teaching contents such as field epidemiological investigation, social health problem investigation, and handling of sudden public health incidents are insufficient, which leads to a lack of training of students’ field investigation ability, comprehensive analysis ability, problem solving ability, and emergency response ability (35). In addition, the executive functions of teaching, research, and practice in China’s public health system belong to the College of Public Health and the Center for Disease Prevention and Control, respectively, which leads to some separation and disconnection between the development of public health teaching and social practice. Undergraduate students majoring in clinical medicine have only arranged internships in various clinical specialties, with few opportunities to participate in public health practice. They lack knowledge and practical skills in public health and have a significant lack of theoretical knowledge and practical training in nosocomial infection, resulting in insufficient ability to deal with public health emergencies and solve practical problems (37). Most of the public health teachers graduated from medical colleges and universities, mostly from school to school, with little practical experience in public health work such as disease prevention and control, health supervision, etc. The lack of practical experience among teachers also restricts the knowledge structure and professional development of students to a certain extent. In addition, there is a lack of necessary practice bases. Moreover, the practice bases are mostly centers for disease prevention and control, health supervision centers, etc., without dedicated personnel responsible for teaching students. The theoretical knowledge of the teachers is insufficient, and they lack the ability to combine theory with practical work, making it difficult to ensure the quality of practice. Finally, the teaching evaluation system is not perfect, and the evaluation of public health education teachers focuses on the publication of scientific research projects and papers, neglecting their teaching ability and performance. The evaluation of students places more emphasis on their exam results, and the assessment of students’ grades is still mainly based on traditional written exams. The evaluation of on-site practical abilities is ignored. There is a lack of professional institutions and personnel for monitoring and evaluating the quality of public health education and teaching. Currently, administrative personnel are mainly engaged in this work, and their level of specialization in monitoring and evaluation is not high (35).

In recent years, some universities have promoted teaching reform and begun to adopt a student-centered approach, carrying out heuristic PBL interdisciplinary organizational teaching with organ systems as modules, expanding teaching venues, and strengthening students’ public health education and community health practices towards the community. But generally speaking, there are still problems such as a single teaching mode, outdated teaching materials, disconnection between curriculum and actual work, insufficient attention from both teaching parties, insufficient faculty, and a single assessment method (34).

### The experience and development of public health education in other countries and weaknesses in the medical education system of China

Public health education in different countries and regions is characteristic of the different cultural backgrounds and economic levels (34). These experiences are worth reviewing to enhance learning and as a reference. This is of great importance to the medical education system in China, which is facing a great challenge as it is reformed.

Public health education in the United States (US) has been established for more than 100 years. The US education system aims to improve the quality of its public health education, which influences countries worldwide. The US has implemented a competency-based educational model. The main component of medical education in the US is based on a competency-based core curriculum. These competencies are achieved through course-based learning objectives. Completion determines the level of graduates’ abilities according to the expected learning objectives, primarily public health needs. The curriculum settings are adjusted as needed to cultivate students’ required competencies and for final evaluations (38).

The core knowledge in public health education is disseminated to the public in the US. By integrating public health knowledge, concepts, and skills into other courses, the Association of American Schools and Programs of Public Health (ASPPH) has cooperated with the Association of American Colleges and Universities (AAC&U) to propose a core curriculum for non-public health students to promote public health education among all college students in the country (39). Having good public health knowledge has become common sense among “educated citizens” of the US (39). The US put forward practice-based public health education, scientific research, and service models. Public health education is based on public health practice, attaching importance to practical training and adapting to professional roles, which have substantially promoted the development of academic and public health practice (40–43).

The US established an accreditation system to ensure the quality of education. Accreditation is an integral part of the system for evaluating teaching quality in higher education. A core principle of professional education institutions is to connect their teaching activities with social purposes. In addition to schools of public health, other colleges and institutions in the US that carry out public health education are included in the accreditation system. Currently, most states in the US require medical practitioners to attend an accredited school of public health or train in an accredited training program (44).

Public financial investment in public health education is increasing in the US. Public finance is the primary sustainable funding source of public health education (38). In the history of public health education in the US, insufficient investment led to problems in health education. However, a depleted and overworked public health workforce has led to an enormous brain drain in public health over the past decade in the US (45).

The educational model of Canada is similar to that of the US, with the same curriculum content but more curriculum categories and class hours. The teaching content emphasizes the mutual penetration and organic combination of basic theory and practice and adopts an equal and interactive teaching form. Students participate in various types of academic activities and prepare regular reports during their probation and internship (46).

Medical colleges in the United Kingdom (UK) integrate clinical work and prevention work, regarding disease prevention as parallel to medical treatment and rehabilitation, and physicians perform public health tasks. The teaching content highlights the cutting-edge nature of knowledge and is closely related to the current needs of public health and preventive medicine. Curriculum setting and teaching in the UK attach great importance to combining these with practical work, cultivating students’ ability to solve practical problems and produce all kinds of talents with practical skills soon after graduation. The teaching methods are diverse, and the curriculum is flexible and not limited by textbooks. Teachers guide and assess students’ learning, focusing on cultivating students’ ability to acquire knowledge independently. The training objectives of students are clear and strict, and the government also strictly supervises the quality of education (34).

Australia adopts Harden’s tricyclic instructional approach, which emphasizes training in attitude and behavior, operational and communication skills, public health safety, and understanding of the changing patterns of health care (34).

Europe and Australia focus more on cultivating “soft skills” in medical practice (34). The European School of Public Health has been affiliated with the medical profession since its inception, which limits its non-medical development in areas such as sociology and health policy (47).

Most institutions in Spain have established unique non-fixed courses in which students have maximum autonomy to participate in their curriculum design through self-directed learning. Schools also adopt the “outcome-based education” model, which determines the evaluation method based on the different abilities of students in their future work (34).

Medical colleges in Japan emphasize multiple forms of medical education and introduce problem-based, site-centered, and case-guided heuristic and discussion teaching methods to achieve the minimization of teaching activities, short-term curriculum arrangement, and diversification of teaching forms (34).

The Korean School of Public Health curriculum plan does not fully consider health care. The teaching process ignores the cultivation of practical skills, implementing teacher-centered didactic education, and the evaluation of students is still based on scores or examinations (34).

According to the experience of public health education in other countries, model of public health education of clinical medicine in China and the current low level of Chinese medical students’ knowledge regarding public health emergencies, we can identify some weaknesses in the medical education system of China.

The traditional education model in China is that the curriculum determines the teaching objectives rather than the expected learning objectives. This means that the public’s health needs cannot be effectively met.

In China, the core curriculum closely related to public health practice in public health education has not yet been established and popularized in medical colleges and universities, let alone among college students, which reflects the fact that these educational institutions do not pay sufficient attention to public health education.

For a long time, medical education in China has mainly adopted traditional theoretical courses to impart knowledge and experience. The teaching method is relatively simple, outdated, and unattractive to younger people. This old style of teaching cannot cultivate students’ ability to analyze and solve practical problems (12, 28).

There is no public health education accreditation system in China for clinical medical students similar to those in other countries. Therefore, the quality of education cannot be guaranteed.

At present, China’s investment in public health education is also far from adequate.

### Recommendations for cultivating medical college students’ ability to deal with public health emergencies in China

In this context, cultivating medical college students’ ability to respond to public health emergencies has become an essential issue in medical education in China and globally. The following suggestions can be used as a reference in China and other countries.

In reviewing the experience of public health education in other countries and the weakness identified in the medical education system of China, we believe that, first, the traditional Chinese educational model must be transformed based on the goals of the curriculum in a competency-based education model. Competency-based (or outcomes-based) education requires the instructor to work backward, from the desired course learning outcomes to the method. Therefore, we need to first define the desired course learning outcomes according to public health needs, and then adjust the curriculum settings, create the content and learning objectives for the course that will yield those desired outcomes, and determine the assessment mechanisms that will supply data on whether those outcomes were met, to train students to achieve these goals. Competency-based approaches lead us more directly to ideas for assessment because the instructional content is explicitly tied to learning outcomes (48).

Second, we should build corresponding public health teaching platforms, such as core curriculum settings and teaching material planning according to the course learning outcomes, that is, health needs. In terms of teaching content, it is suggested that this mainly covers the background knowledge, basic concepts, and theories of public health emergencies, introduction to relevant laws and regulations, professional ethics education, the hierarchical management system, and specific cases of public health emergencies. In addition, students should be systematically and comprehensively introduced to the particular tasks and responsibilities of medical institutions and medical workers in treating public health emergencies (7, 11). We can refer to the core curriculum certified in the US since 2014, including (1) biostatistics; (2) epidemiology; (3) environmental health science; (4) health service management; and (5) social and behavioral sciences.

The importance of teaching methods can also not be ignored. Table 4 shows the preferred training methods of medical college students with knowledge or skills related to public health emergencies. This suggests that colleges and practice bases should diversify the educational forms using various channels and pay attention to the combination of theoretical knowledge and practice. Different methods can be adopted, such as educational media, visiting popular science education sites, networking, lectures, award-winning contests, simulation exercises, case analysis, scenario demonstration, on-site observation, and on-site investigation (21, 24, 26, 28).

Third, public health education can be focused on ideologically, and the public health core curriculum in medical education can be promoted. China should incorporate the core concept of public health into the general education curriculum of medical colleges or universities as soon as possible such that every medical college student can master the basic knowledge, skills, and attitudes of public health. In particular, it is necessary to take adequate measures to organically combine the core public health curriculum with essential medicine and clinical medicine by reforming the curriculum of medical college students. The core curriculum should be taught and examined in compulsory courses. This will be of great help in improving the awareness of medical college students regarding knowledge related to public health emergencies (49).

Fourth, in public health education in China, practical courses should be strengthened to provide more practice opportunities for medical college students. In real-world examples and scenarios, students must be able to apply and practice techniques in each core discipline of public health. With public health practice as the link, we can ensure the quality of public health through teaching, research, and service. The emergency treatment of infectious diseases and public health emergencies is the most critical public health work undertaken by medical institutions. To perform these tasks well, medical institutions rely on timely reporting and handling by medical staff. Therefore, medical college students at the stage of clinical practice, under the instructor’s guidance, can undertake some work such as writing medical records, reporting the epidemic situation of infectious diseases, and other public health monitoring tasks (7, 11). Teaching hospitals should educate students in clinical knowledge and skills and give these students the opportunity to master specific public health work undertaken by medical institutions and how to correctly complete such work as assigned by high-level health administrative departments (11). Students should be taught to consult the literature, capture the latest information in a timely manner, and write papers and reports.

Fifth, we need to implement a public health education certification system and include colleges or universities that carry out public health education-related projects in the accreditation system. The accreditation of public health education in the US has been implemented for nearly 80 years. The development of accreditation standards in public health promotes the development of public health competency in that country. In this regard, China is still in the exploratory stage and is facing the same challenge in terms of improving the quality of public health education.

Finally, public health education needs the guarantee and support of funds. Therefore, we should increase public financial investment in public health education and improve the efficiency of its use. Medical education donations and personal investments are also important sources of funds.

### Looking back to the past, based on the present, the future is full of challenges and hope

Clinical medicine and public health medicine were integrated and inseparable in the past. However, the Rockefeller Foundation’s decision to support the establishment of an independent school of public health in 1916 marked the institutionalization of the division between clinical and public health medicine. In the 1980s, China’s higher education major catalog divided medicine into clinical medicine, basic medicine, and public health and preventive medicine. Since then, public health/preventive and clinical medicine have gradually become separated (11). This kind of medical education system involving the separation of clinical medicine from public health and preventive medicine has prematurely solidified the direction of development among medical college students, resulting in most focusing on individuals rather than groups and emphasizing treatment rather than public health and prevention (11).

In response to this problem, in 2006, the ASPPH and AAC&U reached a consensus that the core curriculum in public health (also known as 101 series courses) should be available in all undergraduate institutions and should fulfill the distribution requirements as part of general education (50, 51). Moreover, the training of students in public health and preventive medicine in the US is based on the exercise of clinical medicine; that is, public health and preventive medical students acquire a clinical medical degree before training in core knowledge and public health skills (52). Therefore, clinical medicine and public health and preventive medicine are well integrated in the US.

In China, however, this problem remains to be resolved. Most medical colleges and universities do not conduct theoretical or practical courses in preventing and responding to public health emergencies. Only a few colleges and universities offer such studies; however, due to the limited class hours, these courses are far from sufficient, and the content is relatively outdated and backward. The assessment of relevant knowledge is carried out in elective rotations for medical college students majoring in non-public health and preventive medicine, which also leads to students attaching little importance to these courses (6). All the above factors lead to an insufficient ability of medical college students to deal with public health emergencies. When the SARS epidemic broke out, medical staff and public health workers did not understand each other’s working methods; these two groups could not communicate and collaborate in a timely manner, which result in heavy losses of health and life (11).

It is imperative to strengthen the reform of public health education for medical college students such that it becomes part of the essential contents of medical education (7, 53). We should help medical college graduates to appreciate the importance of acting effectively in public health emergencies as well as in people’s everyday lives to ensure the health of the nation and countries worldwide. Medical college students should understand that in the future, they must take responsibility for medical responses and public health work in dealing with public health emergencies (11). Strengthening the cultivation of medical college students’ knowledge and ability to prevent and respond to public health emergencies, enhancing their prevention/control awareness and coping ability, and highlighting the leading roles of medical college students in public health emergencies are necessary and vital measures to effectively prevent and control public health emergencies that may be faced in the future (6).

Most medical college students are highly interested in acquiring public health knowledge, especially those who enrolled after the COVID-19 outbreak in 2019, whose understanding and emphasis on public health knowledge have significantly improved. This has laid a good foundation for providing more in-depth education in public health knowledge and responsibilities among medical college students and also reflects that the reform of responsible public health education for medical college students is urgent (11).

Through appropriate, reasonable, and targeted educational reform measures, it is believed that in the future, we can develop and maintain a clinical workforce with the skills and capacities to perform optimally in public health emergencies, to shoulder these vital tasks and minimize the harm caused by such emergencies (7). Achieving this goal can benefit all of society and warrants adequate support.

### Strengths and limitations

For the first time, we systematically reviewed the cognitive status and training needs of medical college students in response to public health emergencies in China. However, several limitations need to be addressed. Firstly, due to the considerable heterogeneity of research design and outcome variables, it was impossible to perform an effective meta-analysis. Secondly, this review excluded studies that did not include clinical medical students, such as those examining the status of nursing students’ ability to respond to public health emergencies. Finally, there may be interference from other related factors. Although the participants in some studies are similar in age, their sex ratios are quite different; moreover, most of the studies did not show this clearly. Nevertheless, this research should be adequate to reflect current situation of insufficient cognition and training needs of medical college students responding to public health emergencies in China.

## Conclusion

This article illustrates the importance of improving medical college students’ education to prevent and deal with public health emergencies. Through systematically reviewing the literature, we found some weaknesses in China’s medical education system that result in the inability of current medical college students to deal with public health emergencies safely and effectively. By considering these weaknesses and summarizing the experience and development of successful public health education in the US and other countries, we put forward some recommendations to improve medical college students’ education in responding to public health emergencies. It is necessary to establish a competency-based educational model, build a core curriculum related to public health, and promote this model in medical colleges and universities. This core curriculum should be based on practice and emphasize practical training. We need to improve the quality of education through a standardized accreditation system. Finally, we should increase public financial investment in public health education and improve the efficiency of its use.

## Author contributions

PY conceptualized this study and made the tables for the manuscript. PY and LZ offered the main direction and significant guidance of this manuscript. XF performed the data analysis and drafted the manuscript with advice from PY. RP and HL revised the manuscript. All authors contributed to the article and approved the submitted version.
